# From Cells to Animals: Connexin43 Suppression Enhances Autophagic Flux to Restore Odontogenesis in Inflamed Dental Pulp

**DOI:** 10.1111/iej.70044

**Published:** 2025-10-05

**Authors:** Peiling Hu, Yingqing Chen, Ping Long, Anni Zhang, Xiaorong Lan, Yuanpei He, Guangwen Li, Shiting Li

**Affiliations:** ^1^ Department of Operative Dentistry and Endodontics, School of Stomatology Southwest Medical University Luzhou China; ^2^ Luzhou Key Laboratory of Oral & Maxillofacial Reconstruction and Regeneration, The Affiliated Stomatological Hospital Southwest Medical University Luzhou China

**Keywords:** autophagic flux, autophagy, connexin43, highly inflammatory conditions, odontogenic differentiation

## Abstract

**Aim:**

Under certain conditions, infection‐induced inflammation may activate reparative processes to form a hard tissue barrier against microbial invasion. Autophagy participates in odontogenic differentiation during inflammation in vitro, but its role in pulp repair remains unclear. This study investigated how autophagy regulated odontogenic differentiation within the inflammatory microenvironment, emphasising the regulatory role of connexin43 (Cx43) on autophagy.

**Methodology:**

Autophagy activation was detected in inflamed pulpal tissues using immunofluorescence (IF). Human dental pulp cells (hDPCs) were stimulated with 0.1/5 μg/mL lipopolysaccharide (LPS). Autophagy dynamics, including autophagic flux, were analysed through western blotting (WB), transmission electron microscopy (TEM), and mRFP‐GFP‐LC3 plasmid transfection. Odontogenic differentiation and mineralisation were assessed using molecular assays and Alizarin Red staining. Pharmacological inhibitors or activators were applied to determine autophagy's role. Cx43 knockdown in hDPCs and a dentine injury model in Cx43 cKO mice were used to validate their effects on autophagy. Results were analysed by two‐way ANOVA.

**Results:**

Autophagy‐related proteins were predominantly localised in the odontoblast layer. As dental pulp infection advanced, concurrent upregulation of LC3 and p62 levels was observed, indicating that autophagy activation occurs in the pulp alongside potential impairment of autophagic flux. 0.1 μg/mL LPS promoted autophagic flux, thereby facilitating odontogenic differentiation and mineralisation, whereas autophagy inhibition attenuated these effects. Conversely, 5 μg/mL LPS induced autophagosome accumulation but blocked autophagic flux, suppressing odontogenic differentiation and mineralisation; however, restoring autophagic flux reversed this inhibition. These data suggested that maintenance of autophagic flux integrity is essential for sustaining odontogenic differentiation capacity under inflammatory stress. Cx43 knockdown under high‐inflammatory conditions rescued autophagic flux and improved differentiation. Similarly, in the dentine injury model of cKO mice, Cx43 deletion attenuated p62 expression while upregulating DSPP expression, accompanied by enhanced tertiary dentine formation beneath the injury site, indicating that blockade of Cx43 promotes autophagic flux to improve pulp repair.

**Conclusion:**

Autophagy regulates both inflammatory responses and repair processes in dental pulp. Specifically, maintaining functional autophagic flux ensures cells adapt to pathological stress while retaining their ability to form mineralised tissue. Cx43 inhibition promotes pulp repair through restoration of autophagic flux under inflammatory conditions, highlighting its potential as a therapeutic target for deep caries by synergistically modulating inflammation and promoting regeneration.

## 1 Introduction

Dental pathologies primarily originate from microbial infections, where pathogens adhere to tooth surfaces, erode enamel and dentine, and subsequently trigger inflammatory responses in pulp tissue to eliminate invading microorganisms. Concurrently, this inflammatory cascade activates odontogenic differentiation of dental pulp cells, facilitating tertiary dentine formation as a defensive barrier against microbial infiltration and preserving the integrity of underlying healthy pulp. Studies in vitro have demonstrated that lipopolysaccharide (LPS) at concentrations of 0.01–1 μg/mL enhances odontogenic differentiation and mineralisation in various dental stem cells via Extracellular signal‐Regulated Kinase (Erk)/p38 Mitogen‐Activated Protein Kinase (MAPK) and Phosphatidylinositol 3‐Kinase/Protein Kinase B (PI3K/Akt) signalling pathways (He et al. 2015; Wang et al. 2015; Liu et al. 2019), suggesting that low‐grade inflammation promotes pulp tissue repair. However, the mechanisms governing pulp repair under persistent low‐grade or high‐intensity inflammatory conditions remain poorly elucidated.

Autophagy, a highly conserved lysosomal degradation pathway in eukaryotes, selectively removes damaged organelles, misfolded proteins, and intracellular pathogens through a multi‐step process (Levine and Kroemer 2019). This process initiates with the formation of double‐membrane autophagosomes derived from the rough endoplasmic reticulum, which subsequently fuse with lysosomes to degrade encapsulated contents, termed autophagic flux (Mizushima and Komatsu 2011; Parzych and Klionsky 2014; Zhou et al. 2012). In addition to maintaining cellular balance, autophagy modulates immune and inflammatory responses by balancing beneficial and harmful outcomes. This dual regulation impacts diseases such as infections, autoimmune disorders, and chronic inflammation (Deretic 2021; Levine et al. 2011). Moreover, emerging evidence highlights its involvement in tissue repair (Boya et al. 2018), as keratinocyte autophagy accelerates wound healing by activating keratinocytes and fibroblasts (Qiang et al. 2021). In dental contexts, autophagy marker microtubule‐associated protein 1 light‐chain 3 (LC3) has been detected in odontoblast layers of carious and pulpitic human teeth (Wang et al. 2016), implicating autophagy in pulpal infection and inflammation. Notably, LPS‐induced autophagy in pre‐odontoblastic cells exhibits dual roles: sustaining cell survival during early inflammation while promoting apoptosis at later stages (Pei et al. 2015). Furthermore, autophagy enhances odontogenic differentiation under inflammatory conditions by suppressing phosphorylated nuclear factor‐κB (NF‐κB) signalling (Pei et al. 2016), potentially through augmented autophagosome‐lysosome fusion to facilitate autophagic flux (Cho et al. 2021).

The regulatory network of autophagy is intricate in inflammation and repair. Connexin 43 (Cx43), a gap junction protein forming gap junction (GJ) intercellular channels or hemichannels, modulates physiological and pathological processes through direct substance exchange (Rouhani et al. 2023; Lim et al. 2021; Martins‐Marques et al. 2019). Our previous investigations revealed that Cx43 participates in pulpal infection and inflammation, with its inhibition attenuating LPS‐induced inflammatory responses in human dental pulp cells (hDPCs) by blocking damage‐associated molecular pattern (DAMP) release through hemichannel closure (Hu et al. 2024; Long et al. 2024). Additionally, Cx43 potentiates Ca^2+^‐induced the odontogenic differentiation of hDPCs in vitro, likely via gap junction‐mediated communication (Li et al. 2015). Intriguingly, Cx43 interacts with autophagy‐related proteins to regulate autophagosome formation (Iyyathurai et al. 2016). Furthermore, gap junction‐mediated communication by Cx43 modulates autophagic flux, as evidenced by its role in modulating cadmium‐induced autophagic flux in Buffalo rat liver cells (Zou et al. 2020; Duan et al. 2023). These findings position Cx43 as a potential nexus between inflammation, autophagy, and tissue repair in dental pulp.

This study investigated autophagy activation patterns in human third molars with varying caries depths (superficial, intermediate/deep) and pulpitis, while delineating the functional significance of autophagic flux in odontogenic differentiation of hDPCs under inflammatory conditions. Furthermore, we explored the regulatory interplay between Cx43 and autophagy during inflammation‐driven dentine formation, aiming to elucidate novel mechanisms underlying pulp repair in pathological states.

## 2 Materials and Methods

**FIGURE 1:**
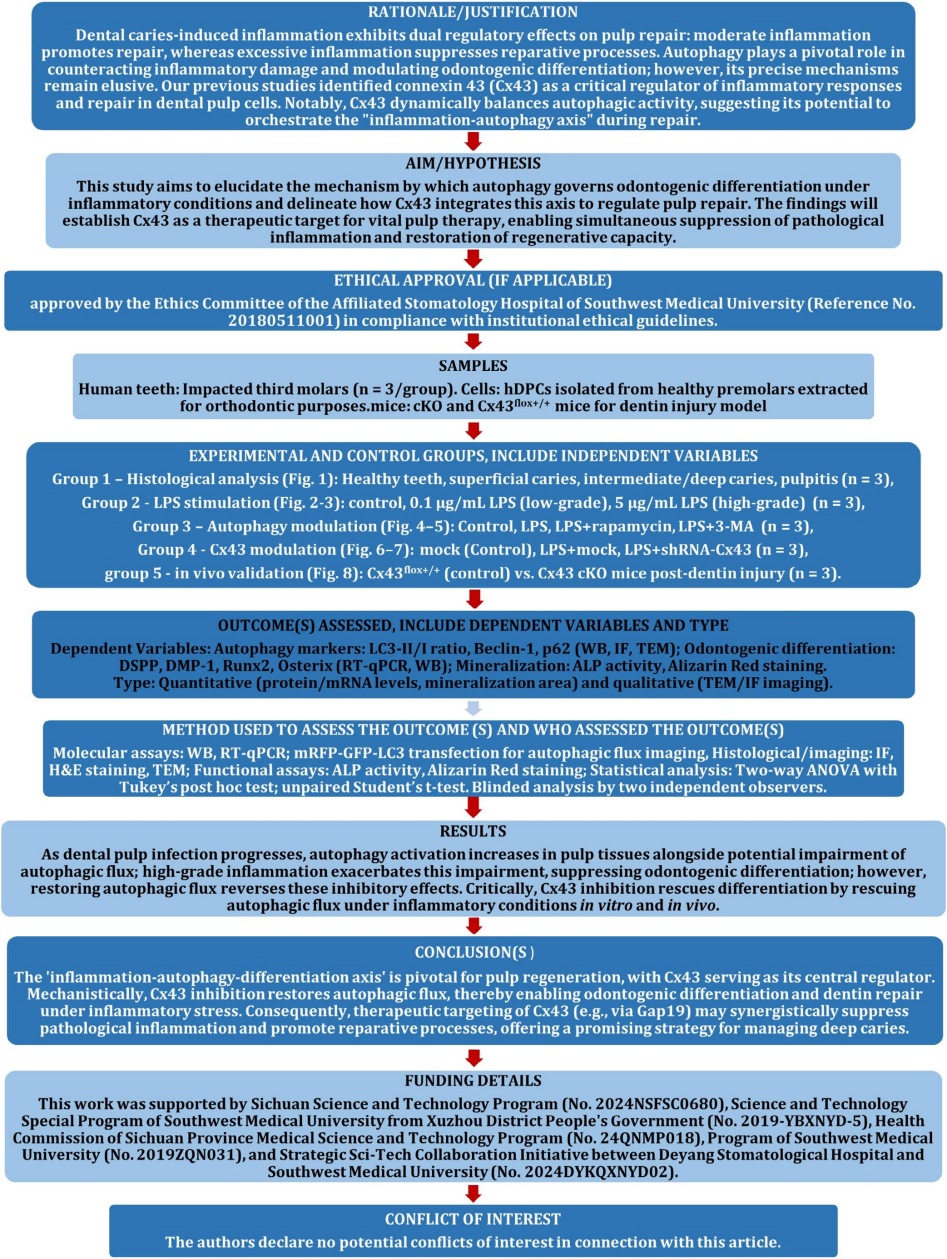
Flowchart of the experimental design for this study.

The manuscript of this laboratory study has been written according to Preferred Reporting Items for Laboratory studies in Endodontology (PRILE) 2021 guidelines. The study design is illustrated by a PRILE flowchart in Figure 1. All experimental procedures involving human cells, animal subjects, and human dental specimens were approved by the Ethics Committee of the Affiliated Stomatology Hospital of Southwest Medical University (Reference No. 20180511001) in compliance with institutional ethical guidelines.

### 2.1 Cell Culture

Healthy and intact premolars were extracted for orthodontic treatment purposes. All patients provided informed consent. hDPCs were isolated and prepared as described previously (Huang et al. 2006). Cells were maintained in α‐minimal essential medium (α‐MEM; Thermo Fisher Scientific Inc., USA) with 10% fetal bovine serum (FBS; Thermo Fisher) and 1% penicillin/streptomycin (Thermo Fisher) at 37°C in a humidified atmosphere of 95% air and 5% CO_2_. Cells from passages 3 through 10 were used for experiments. hDPCs were cultured with odontogenic induction medium (OI) consisting of α‐MEM containing 10% FBS, 50 mg/mL ascorbic acid, 10 mM sodium β‐glycerophosphate, and 10 nM dexamethasone (Sigma‐Aldrich, USA) (Pei et al. 2016), with or without 0.1/5 μg/mL *Escherichia coli* ‐derived LPS (Cat. No. L2630, Sigma) for 7, 14, and 21 days, replacing the medium every 3 days. The cells were divided into three experimental groups: control group (OI medium without LPS stimulation), 0.1 μg/mL LPS‐treated group, and 5 μg/mL LPS‐treated group.

### 2.2 Human Teeth Samples Histological Analysis

Human third molars were collected from patients aged 20–40 years under strict exclusion criteria (incomplete root development, cracked teeth). Based on clinical and radiological examinations, they were categorised into four groups: (1) intact healthy teeth (control), (2) superficial caries, (3) intermediate/deep caries, and (4) irreversible pulpitis (*n* = 3 per group). Informed consent was obtained from all participants. The teeth were fixed in 4% paraformaldehyde solution for 48 h, followed by decalcification in 17% ethylenediaminetetraacetic acid (EDTA) (pH 7.4) at room temperature for 16 months. For light microscope analyses, tissues were embedded in paraffin. Thin sections (5 μm) were stained with haematoxylin and eosin or processed for immunofluorescence analysis.

### 2.3 Animals/Animal Models

To establish conditional knockout (cKO) mice with Cx43 deletion specifically in dental pulp cells, we generated dentine sialophosphoprotein (DSPP) Promoter‐Cre; Cx43^flox+/+^ mice using the Cre/LoxP recombination system. Cx43^flox+/−^ heterozygous mice and DSPP pro‐CreERT mice were obtained from Cyagen Biosciences (USA). Briefly, male Cx43^flox+/−^ heterozygous mice were crossed with wild‐type C57BL/6 (B6) mice to generate Cx43^flox+/+^ littermates, which served as controls. Subsequently, male Cx43^flox+/−^ heterozygous mice were crossed with CreERT^+^ mice to produce CreERT^+^; Cx43^flox+/−^ offspring. These mice were intercrossed to obtain CreERT^+^; Cx43^flox+/+^ progeny. To induce Cx43 deletion in mouse dental pulp cells, 4‐week‐old CreERT^+^; Cx43^flox+/+^ mice received intraperitoneal tamoxifen (Cat. No. T5648; Sigma) administration (75 mg/kg) for five consecutive days. Detailed results of cKO mouse generation and genotypic validation were provided in the Supporting Information.

### 2.4 Dentine Injury Model

Dentine injury was induced in the first maxillary molars of 4‐week‐old Cx43^flox+/+^ (control) and cKO mice, following a standardised mechanical drilling protocol as previously described (Zhao et al. 2018). Mice were anaesthetised via intraperitoneal injection of ketamine (80 mg/kg) and xylazine (8 mg/kg). A 0.3‐mm‐diameter round bur (Cat. No. E0123; Dentsply Maillefer, USA) mounted on a high‐speed dental handpiece was used to create standardised cavities on the occlusal surface of the first maxillary molars. To minimise direct pulp exposure, drilling was primarily directed toward the dental fossa. However, due to the anatomical complexity of mouse molars (including multiple cusps, limited tooth size, and bur dimensions), minor enamel removal near or at the cusp tips was unavoidable. Mice were euthanised at postoperative days 1, 7, and 14. Maxillae were dissected and fixed in 4% paraformaldehyde for 48 h at 4°C, followed by decalcification in 17% EDTA (pH 7.4) for 3 months with solution replacement every 3 days. Decalcified specimens were dehydrated through a graded ethanol series, embedded in paraffin, and sectioned at 5‐μm thickness for histological analysis.

### 2.5 Short Hairpin RNA Gene Knockdown

To inhibit Cx43 expression in cells, hDPCs were transfected with lentiviral particles carrying short hairpin RNA targeting Cx43 (shRNA‐Cx43) or a non‐targeting control (shRNA‐mock) at a multiplicity of infection (MOI) of 40, as previously described (Long et al. 2024).

### 2.6 Real‐Time Quantitative Polymerase Chain Reaction

Total RNA was extracted from cultured cells and first‐strand cDNA was synthesised from 0.5 μg of RNA using an Advantage RT‐for‐PCR Kit (Takara Bio Inc., JP). Gene expression was analysed by real‐time quantitative polymerase chain reaction (RT‐qPCR) with an Applied Biosystems (Thermo Fisher) 7500 Real‐time PCR System in 20 μL of Titanium One‐Step RT‐PCR Kit (Takara) containing 10 mmol/L each of the specific primers (Table 1). The results were standardised to the housekeeping gene GAPDH and expressed as relative mRNA levels.

**TABLE 1:** Gene‐specific primers for PCR amplification.

Gene	Primers (5′–3′)
*Dentine sialophosphoprotein (DSPP)*	F: CTGGTGCATGAAGGTGATAGAG
	R: TCCTACTTCTGCCCACTTAGA
*Dentine matrix protein‐1 (DMP‐1)*	F: GTGAGTGAGTCCAGGGGAGATA
	R: ATTTTGAGTGGGAGAGTGTGTGC
*Osterix (Osx)*	F: CCTCCTCAGCTCACCTTCTC
	R: GTTGGGAGCCCAAATAGAAA
*Runt‐related transcription factor 2 (Runx2)*	F: CCAACCCACGAATGCACTATC
	R: TAGTGAGTGGTGGCGGACAT
*Connexin43 (Cx43)*	F: CTGGGGGTGTATGGGGTAGA
	R: TTCTTAGGGGTGTTTGCGGG
*GAPDH*	F: CTCTGGTAAAGTGGATATTGT
	R: GGTGGAATCATATTAGAACA

### 2.7 Western Blotting

Total protein was isolated in RIPA buffer (Sigma), separated by SDS‐PAGE, and transferred to PVDF membranes (EMD Millipore/Merck KGaA, USA). Subsequently, the membranes were incubated with a blocking buffer containing 5% non‐fat dried milk in TBS Tween‐20 buffer and cultured at 4°C overnight with antibodies against LC3 (Cat. No. ab192890, Abcam, UK; 1:2000), Beclin‐1 (Cat. No. 11306‐1‐AP, Proteintech, USA; 1:5000), sequestosome‐1/SQSTM1 (p62; Cat. No. 18420‐1‐AP, Proteintech; 1:2000), DSPP (Cat. No. bs‐8557R, Bioss, CN; 1:1000), dentine matrix protein‐1 (DMP‐1; Cat. No. bs‐25502R, Bioss; 1:1000), Osterix (Osx; Cat. No. ab209484, Abcam; 1:1000), runt‐related transcription factor 2 (Runx2; Cat. No. 20700‐1‐AP, Proteintech; 1:500), Cx43 (Cat. No. ab11370, Abcam; 1:2000), and GAPDH (Cat. No. 10494‐1‐AP, Proteintech; 1:10 000), followed by incubation with secondary antibodies at 37°C for 2 h. Blots were developed with a Supersignal West Pico chemiluminescent substrate kit (Thermo Fisher) with GAPDH serving as a loading control. Quantification of protein bands was performed using ImageJ software (NIH, USA).

### 2.8 Haematoxylin and Eosin Staining

Deparaffinised sections were rehydrated through graded ethanol and stained with haematoxylin (Beyotime Biotechnology Co. Ltd., CN) for 5 min, followed by differentiation in acid alcohol and bluing in tap water. Sections were counterstained with eosin (Beyotime) for 1 min, dehydrated in graded ethanol, cleared in xylene, and mounted with neutral resin. Tissue sections were digitally scanned using a KF‐PRO‐020‐HI high‐resolution slide scanner (KFBIO, CN) for microscopic observation and analysis.

### 2.9 Immunofluorescence

Tissue slices or cell samples were permeabilized with 0.1% Triton X‐100 for 15 min and blocked with 1% bovine serum albumin (BSA) in phosphate buffered saline (PBS) for 1 h. Then, the sections were incubated with Cx43 (1:50), DSPP (1:200), LC3 (1:500), and p62 (1:200) primary antibodies at 4°C overnight. The next morning, the sections were washed three times with tris buffered saline (TBS) followed by incubation with ALEXAFluor 488/647‐conjugated secondary antibodies (Cat. No. ab150077/ab150115, Abcam; 1:100). The nuclei were counterstained with DAPI (Sigma). The sections were then analysed by fluorescence microscopy using a LEICA DM4000B microscope equipped with a Photometrics CoolSnap1 camera and software (Leica, DE).

### 2.10 Transmission Electron Microscopy

Freshly harvested cell specimens were fixed in 2.5% glutaraldehyde (in 0.1 M phosphate buffer, pH 7.4) at 4°C for 12 h, followed by post‐fixation in 1% osmium tetroxide for 2 h. Samples were then dehydrated through a graded ethanol series (50%, 70%, 90%, and 100% ethanol, 15 min each) and embedded in Epon 812 resin. Polymerisation was performed at 60°C for 48 h. Ultrathin sections (80 nm) were cut using a diamond knife on a Leica Ultracut UCT ultramicrotome and mounted on 200‐mesh copper grids. Sections were stained with 2% uranyl acetate in methanol for 20 min (protected from light). Imaging was performed using a Hitachi HT7800 transmission electron microscope operated at 80 kV. Images were captured at magnifications of ×5000 to ×50 000 using a Gatan OneView CMOS camera.

### 2.11 Transient Transfection of mRFP‐GFP‐LC3 Plasmid

The mRFP‐GFP‐LC3 plasmid was obtained from GeneChem Co. Ltd. (CN). hDPCs were seeded in 12‐well plates and transfected with the mRFP‐GFP‐LC3 plasmid for 8 h. Subsequently, the medium was replaced with complete culture medium. Cells were fixed with 4% paraformaldehyde for 30 min and counterstained with DAPI for 10 min. Autophagic flux was analysed by fluorescence microscopy, monitoring the distribution and alteration of mRFP‐GFP‐LC3B fluorescent signals. Autophagosomes (characterised by yellow puncta, GFP^+^/RFP^+^) and autolysosomes (characterised by red puncta, GFP^−^/RFP^+^) were quantified in individual cells with ImageJ software (NIH).

### 2.12 Alkaline Phosphatase Activity Assay

The alkaline phosphatase (ALPase) activity of cells was determined using an alkaline phosphatase assay kit (Beyotime) according to the manufacturer's protocol. hDPCs cultured for 7 days were fixed with 4% paraformaldehyde at room temperature for 30 min. Subsequently, a 5‐bromo‐4‐chloro‐3‐indolyl phosphate (BCIP)/nitro blue tetrazolium (NBT) solution was added to each well and incubated for 5 min at room temperature for the chromogenic reaction. After washing, stained cells were observed under a stereomicroscope (Model: SMZ800N; Nikon, JP). For quantitative analysis of BCIP/NBT staining, images were analysed with ImageJ software (NIH).

### 2.13 Alizarin Red Staining

Cells cultured for 21 days were fixed with 4% paraformaldehyde at room temperature for 30 min. Fixed cells were then stained with 40 mM Alizarin Red (Cyagen, USA; pH 4.2) for 10 min at room temperature. Following thorough washing, mineralisation nodules were visualised and imaged using a stereomicroscope (Nikon). For quantitative analysis, stained areas were measured using ImageJ software (NIH).

### 2.14 Statistical Analysis

All data were presented as the mean ± standard deviation (SD). Prior to statistical analysis, normality was assessed using the Shapiro–Wilk test, and homogeneity of variances was evaluated via Levene's test. All statistical analyses were performed using GraphPad Prism v9.0 (GraphPad Software; GraphPad Software Inc., USA), including Student's *t*‐test (unpaired) and two‐way ANOVA with Tukey's post hoc test. All experiments were independently repeated in triplicate. *p* < 0.05 was regarded as statistically significant.

## 3 Results

### 3.1 Autophagy in Inflamed Dental Pulp

**FIGURE 2:**
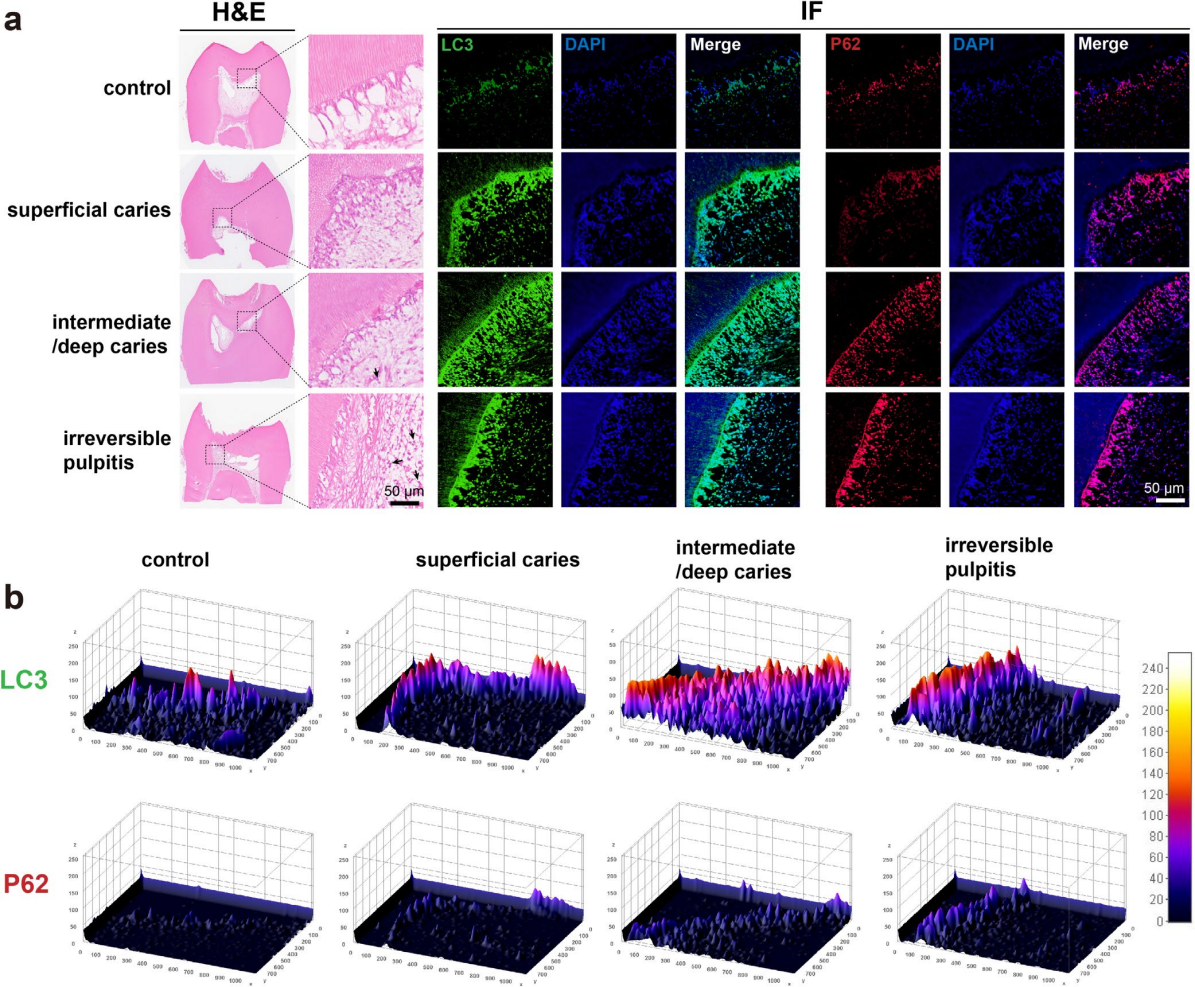
Autophagy in dental pulp tissues. (a) LC3 and p62 immunofluorescence staining in healthy and inflamed dental pulp of human third molars. (b) Quantitative heatmap analysis of fluorescence intensity generated with ImageJ. Black arrows: Inflammatory cell infiltration; *n* = 3 replicates per group.

LC3, a key autophagy‐related gene implicated in autophagosome formation (Mizushima et al. 2010; Castillo et al. 2013), and the reduction of p62, a phenomenon associated with enhanced autophagic flux (Yoshii and Mizushima 2017; Castillo et al. 2013), constitute two mechanistically distinct yet interconnected autophagy markers. As illustrated in Figure 2a, haematoxylin and eosin staining demonstrated marked inflammatory cell infiltration (black arrows) subjacent to the lesion, which intensified with infection severity. IF analysis revealed minimal co‐localization of LC3 and p62 immunoreactivity within the odontoblast layer of intact, healthy dental specimens (control group). Notably, progressive microbial infiltration induced spatial expansion of both biomarkers' expression patterns, exhibiting extensive distribution across the odontoblast layer, sub‐odontoblastic areas, and certain regions within the pulp proper.

Quantitative analysis using ImageJ revealed a progressive increase in both the distribution area and expression levels of LC3 and p62 with disease severity (Figure 2b), indicating a positive correlation between infection severity and immunoexpression intensity of these markers. These findings collectively suggest that: (1) autophagic machinery becomes activated in pulpal tissues during infectious and inflammatory processes; (2) escalated pathogenic challenge promotes autophagosome biogenesis as evidenced by LC3 accumulation; while (3) paradoxical p62 accumulation despite enhanced autophagosome formation implies potential impairment of autophagic flux completion, possibly indicating lysosomal dysfunction or defective autophagosome–lysosome fusion mechanisms under sustained or highly inflammatory conditions.

### 3.2 High‐Grade Inflammation Promoted Autophagosome Formation While Impairing Autophagic Flux in hDPCs

**FIGURE 3:**
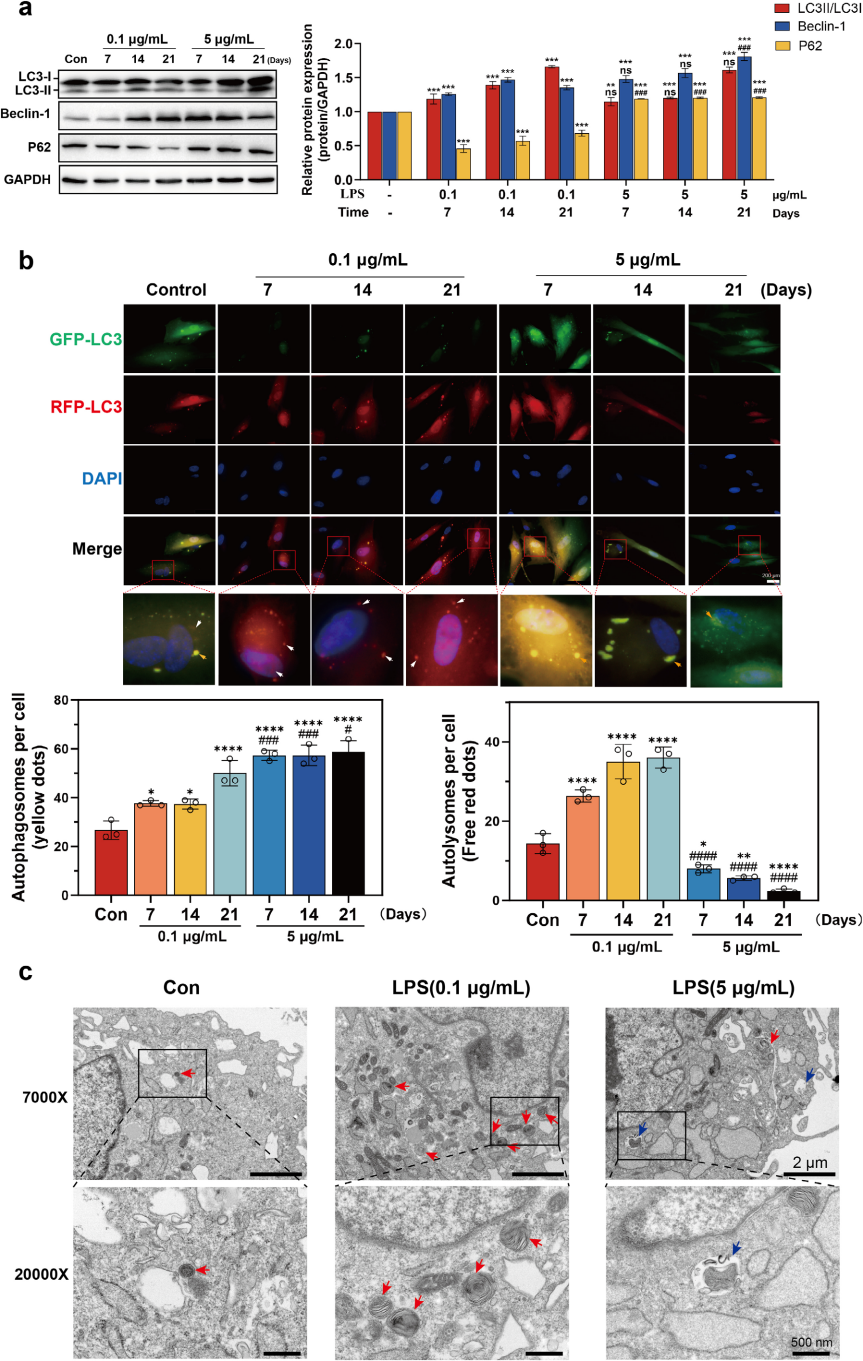
The effect of LPS on autophagy and autophagic flux in hDPCs. hDPCs were stimulated with OI + 0.1 or 5 μg/mL LPS for 7, 14, and 21 days. (a) Representative WB analysis of LC3II/I ratios, Beclin‐1, and p62 protein levels. Band intensities were quantified using ImageJ software. (b) Fluorescence microscopy images of hDPCs transfected with mRFP‐GFP‐LC3. Red puncta (white arrows) represent autolysosomes, while yellow puncta (yellow arrows) indicate autophagosomes. Quantification of puncta per cell using ImageJ. (c) TEM analysis of autophagic ultrastructure after 7‐day OI + LPS stimulation. Autolysosomes (red arrows) and autophagosomes accumulation (blue arrows) are indicated. *p < 0.05, **p < 0.01, ***p < 0.001, ****p < 0.0001 versus control; ^#^ *p* < 0.05, ^###^ *p* < 0.001, ^####^ *p* < 0.0001 versus 0.1 μg/mL LPS group.

Beclin‐1 serves as a critical regulator of autophagosome formation (Mizushima et al. 2010; Castillo et al. 2013). To investigate the dynamic alterations in autophagy and autophagic flux under inflammatory conditions in hDPCs, we initially analysed the protein expression profiles of LC3, Beclin‐1, and p62 via WB. The results demonstrated that 0.1 μg/mL LPS treatment significantly upregulated LC3‐II/I ratios and Beclin‐1 protein levels while downregulating p62 expression across multiple time points compared to the control group (Figure 3a). These observations suggest that low‐grade inflammation enhances autophagic initiation and potentially promotes autophagic flux in hDPCs. In contrast, 5 μg/mL LPS exposure similarly elevated LC3‐II/I ratios and Beclin‐1 expression but paradoxically increased p62 accumulation relative to both control and low‐concentration LPS groups (Figure 3a), suggesting a potential impairment of autophagic flux despite persistent autophagic activation under high‐grade inflammatory stimulation.

To further validate these findings, we employed GFP‐RFP‐LC3 dual fluorescence plasmid transfection to monitor autophagic progression. Autophagosomes (yellow puncta) and autolysosomes (red puncta) were quantified through fluorescent microscopy. Compared to controls, 0.1 μg/mL LPS treatment significantly increased red puncta density (white arrows, Figure 3b) in hDPCs, confirming enhanced autophagosome–lysosome fusion and autophagic flux completion. Conversely, 5 μg/mL LPS exposure elevated yellow puncta count (yellow arrows) while reducing red puncta formation compared to the low concentration group (Figure 3b), demonstrating impaired lysosomal fusion capacity under high‐grade inflammation.

Complementary ultrastructural analysis via TEM after 7‐day LPS exposure in hDPCs revealed distinct autophagic morphologies. The 0.1 μg/mL LPS group exhibited increased autolysosome formation (indicated by red arrows), whereas 5 μg/mL LPS promoted autophagosome accumulation (blue arrows) with suppressed autophagosome‐lysosome fusion (Figure 3c), consistent with immunofluorescence observations. Collectively, these multimodal analyses establish a concentration‐dependent dual regulatory effect of inflammatory stimulation: low‐intensity inflammation facilitates complete autophagic flux through coordinated autophagosome formation and lysosomal degradation, while high‐intensity inflammation induces autophagic initiation but disrupts autophagic flux completion through impaired lysosomal fusion.

### 3.3 High‐Grade Inflammation Suppressed Odontogenic Differentiation and Mineralisation in hDPCs

**FIGURE 4:**
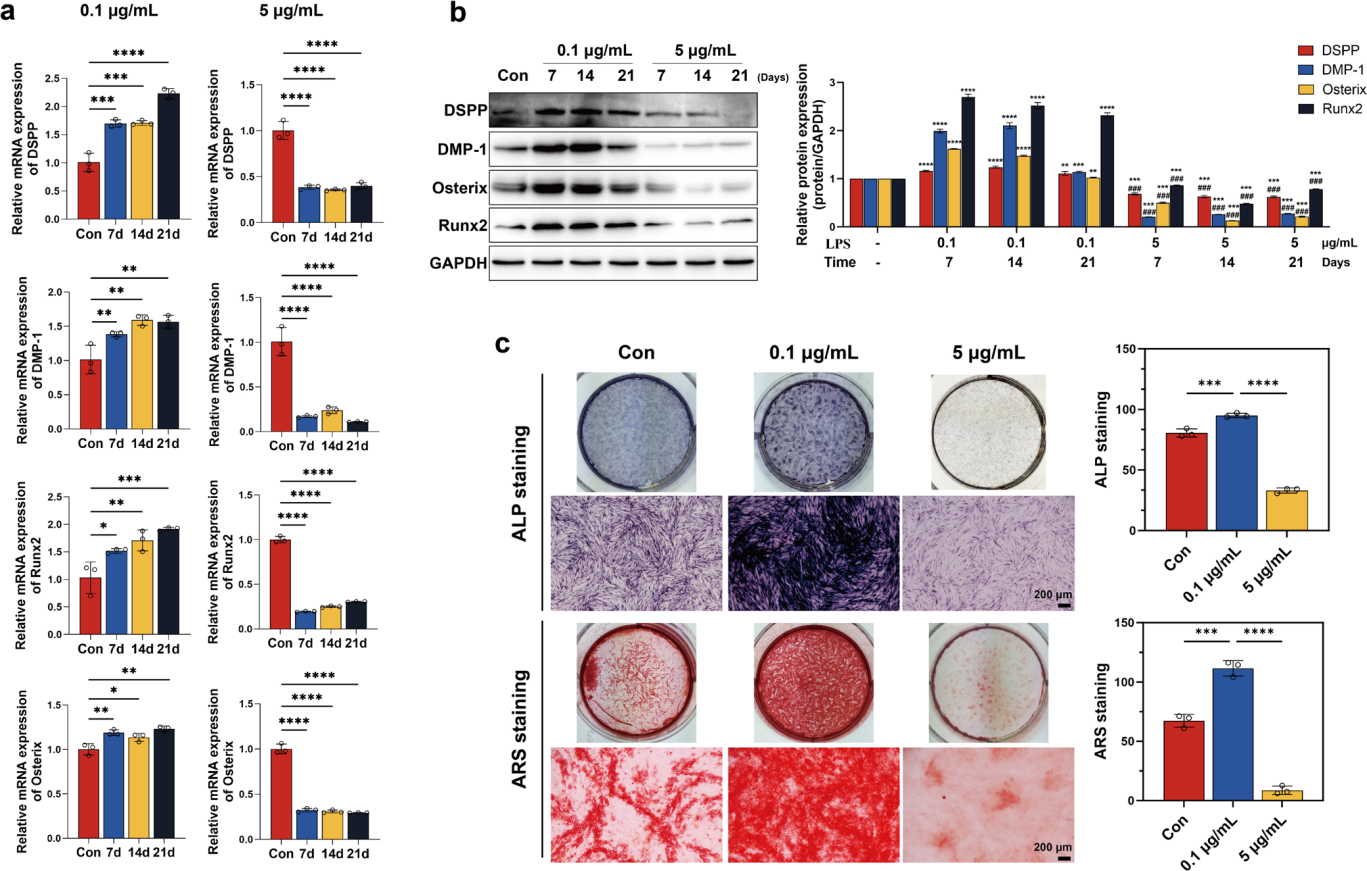
The effect of LPS on odontogenic differentiation of hDPCs. hDPCs were stimulated with OI + 0.1 or 5 μg/mL LPS for 7, 14, and 21 days. (a) RT‐qPCR analysis of the mRNA levels of DSPP, DMP‐1, Runx2, and Osterix. (b) WB analysis of corresponding protein expression. (c) ALP activity (day 7) and Alizarin Red‐stained mineralised nodules (day 21). Quantification with ImageJ. * *p* < 0.05, **p < 0.01, ***p < 0.001, ****p < 0.0001 in (a, c); **p < 0.01, ***p < 0.001, ****p < 0.0001 versus control in (b); ^###^ *p* < 0.001 versus 0.1 μg/mL LPS group in (b).

LPS, a predominant pathogen‐associated molecular pattern in gram‐negative bacterial cell walls, is clinically associated with progressive carious lesions and pulpitis pathogenesis, triggering innate immune responses in dental pulp tissues (Wang et al. 2015). Previous studies have demonstrated that LPS at the appropriate concentration may enhance the proliferation capacity and osteo/odontogenic differentiation potential of dental stem cells (Huang et al. 2015; Liu et al. 2019). To systematically evaluate the dose‐dependent effects of inflammatory stimulation on differentiation processes, we quantified the expression levels of odontogenic biomarkers—DSPP and DMP‐1 (Hanks et al. 1998), along with key transcription factors Runx2 and Osx (Chen et al. 2009), in hDPCs exposed to graded LPS concentrations (0.1 vs. 5 μg/mL). RT‐qPCR analysis revealed that 0.1 μg/mL LPS significantly elevated the mRNA levels of DSPP, DMP‐1, Runx2, and Osx in hDPCs across multiple time points compared to the control (Figure 4a), a trend corroborated by corresponding protein elevation (Figure 4b). In contrast, 5 μg/mL LPS not only abolished these pro‐differentiation effects but also suppressed both mRNA and protein expression below baseline levels (Figure 4a,b).

Functional assays further demonstrated that 0.1 μg/mL LPS enhanced ALP activity, whereas 5 μg/mL inhibited it (Figure 4c). Alizarin Red S staining confirmed that low‐grade inflammation promoted mineralised nodule formation, while high‐grade inflammation suppressed mineralisation (Figure 4c). These data establish that sustained high‐intensity inflammatory signalling disrupts the reparative odontogenic programme in hDPCs, suggesting a mechanistic link between uncontrolled pulp inflammation and impaired dentine regeneration.

### 3.4 Enhancement of Autophagic Flux Facilitated Odontogenic Differentiation and Mineralisation in hDPCs Under High‐Inflammatory Conditions

Emerging evidence underscores the regulatory role of autophagy in odontogenic differentiation under physiological and pathological conditions, with autophagic flux serving as a pivotal mediator of this process (Pei et al. 2016; Zhan et al. 2020). However, our experimental data revealed that high‐concentration LPS significantly suppressed both autophagic flux and odontogenic differentiation in hDPCs. To delineate the mechanistic interplay between autophagy and odontogenic differentiation under inflammatory stress, we pharmacologically induced autophagy using rapamycin (Fu, Zhang, et al. 2024)—a mechanistic target of rapamycin (mTOR) inhibitor that initiates autophagosome formation and enhances lysosomal fusion—prior to 5 μg/mL LPS exposure.

**FIGURE 5:**
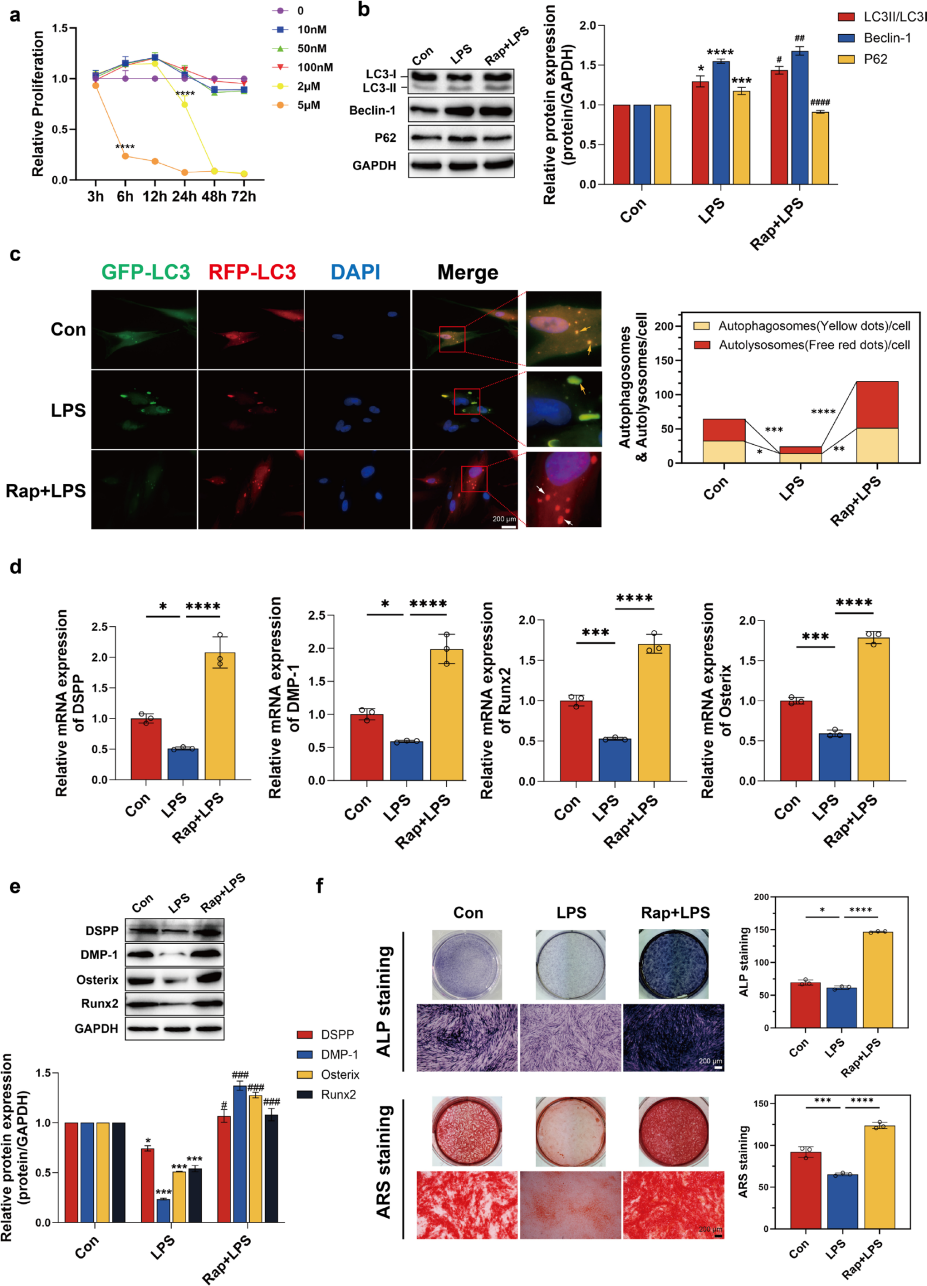
Rapamycin restores autophagic flux and rescues LPS‐impaired odontogenic differentiation. (a) CCK‐8 viability assays to determine optimal rapamycin concentration (100 nM, 24 h pretreatment). hDPCs were pretreated with rapamycin for 24 h before OI + 5 μg/mL LPS stimulation. (b) WB analysis of LC3II/I ratio, Beclin‐1, and p62. (c) mRFP‐GFP‐LC3 fluorescence imaging showing autolysosome (white arrows) and autophagosome (yellow arrows). (d–e) RT‐qPCR and WB analyses of odontogenic markers. (f) ALP activity and mineralisation.*p < 0.05, ***p < 0.001, ****p < 0.0001 versus control in (b, e); ^#^ *p* < 0.05, ^##^ *p* < 0.01, ^###^ *p* < 0.001, ^####^ *p* < 0.0001 versus 5 μg/mL LPS group in (b, e); *p < 0.05, **p < 0.01, ***p < 0.001, ****p < 0.0001 in (c, d, f).

The optimal rapamycin concentration (100 nM, 24‐h treatment) was determined via Cell Counting Kit‐8 (CCK‐8; Beyotime) viability assays (Figure 5a). Immunoblot analysis demonstrated that rapamycin pretreatment in LPS‐stimulated hDPCs markedly upregulated the LC3II/I ratio and Beclin‐1 protein level while downregulating p62 expression (Figure 5b), confirming enhanced autophagic flux. Corroborating this, GFP‐RFP‐LC3 dual fluorescence imaging revealed a pronounced increase in autolysosome formation (quantified by red puncta, white arrows) in rapamycin‐treated cells compared to the LPS‐only group (Figure 5c). These findings collectively indicate that rapamycin not only activates autophagy but restores functional autophagic flux in hDPCs under high‐inflammatory conditions.

Notably, rapamycin‐mediated autophagic flux potentiated the odontogenic differentiation of hDPCs, as evidenced by elevated mRNA and protein expression levels of key markers (DSPP, DMP‐1, Osx, and Runx2) in LPS‐stimulated cells (Figure 5d,e). Extended mineralisation assays further demonstrated that rapamycin significantly augmented ALP activity at day 7 and accelerated extracellular matrix mineralisation at day 21 in LPS‐challenged hDPCs (Figure 5f).

**FIGURE 6:**
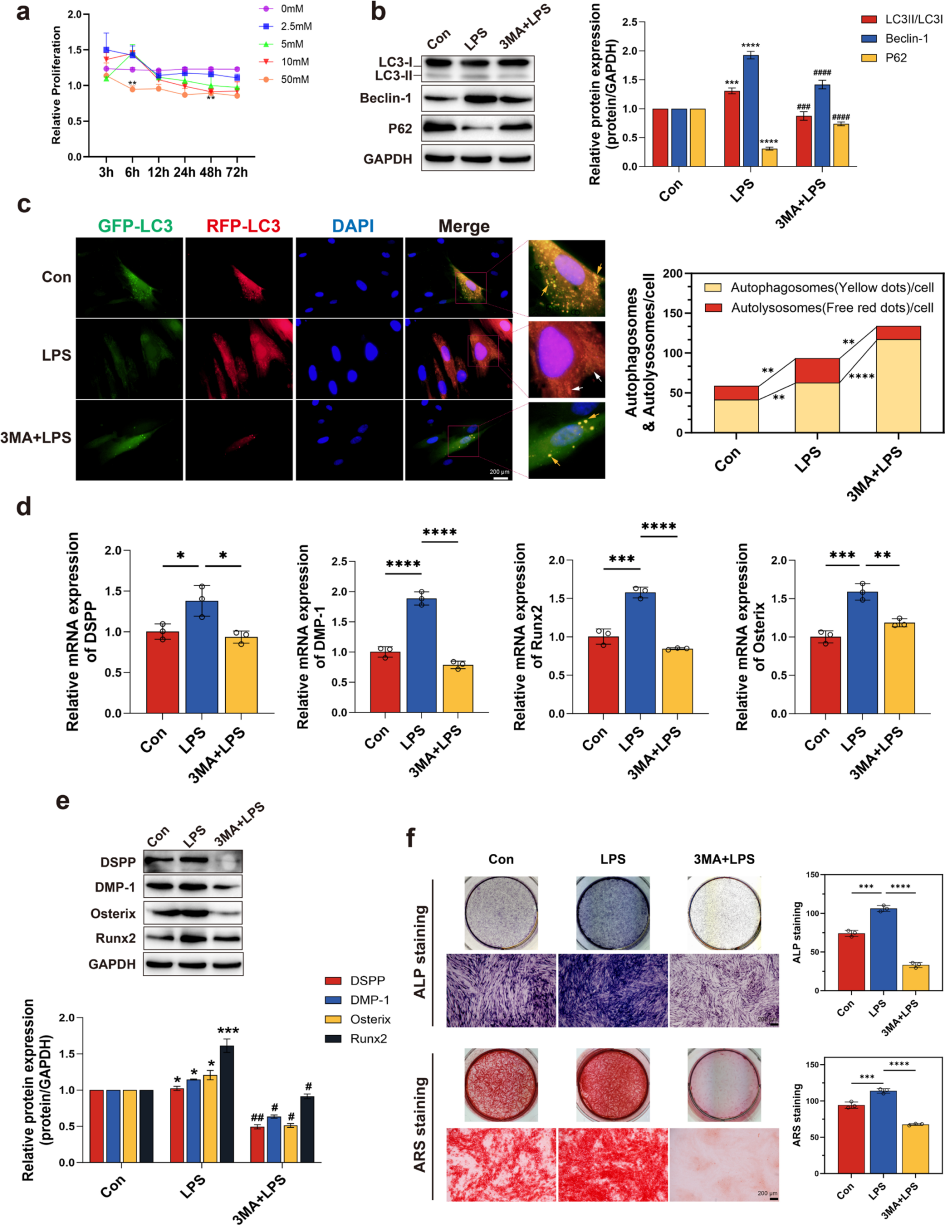
3‐MA inhibits autophagic flux and attenuates LPS‐enhanced odontogenic differentiation. (a) CCK‐8 assays for 3‐MA optimization (5 mM, 24 h pretreatment). hDPCs were pretreated with 3‐MA for 24 h before OI + 0.1 μg/mL LPS stimulation. (b) WB analysis of LC3II/I ratio, Beclin‐1, and p62. (c) mRFP‐GFP‐LC3 fluorescence imaging and quantitative analysis. (d, e) RT‐qPCR and WB analyses of odontogenic markers. (f) ALP activity and mineralisation. *p < 0.05, ***p < 0.001, ****p < 0.0001 versus control in (b, e); ^#^ *p* < 0.05, ^##^ *p* < 0.01, ^###^ *p* < 0.001, ^####^ *p* < 0.0001 versus 0.1 μg/mL LPS group in (b, e); **p < 0.01, ***p < 0.001, ****p < 0.0001 in (c, d, f).

To validate the causal role of autophagic flux, we inhibited autophagy using 3‐methyladenine (3‐MA; Liu et al. 2021; 5 mM, 24‐h pretreatment) prior to low‐dose LPS stimulation (0.1 μg/mL). CCK‐8 assays confirmed the non‐cytotoxic 3‐MA concentration (Figure 6a). WB analysis revealed that autophagy inhibition by 3‐MA attenuated LPS‐induced LC3II/I ratio and Beclin‐1 protein level, while elevating p62 accumulation (Figure 6b). These biochemical changes were corroborated by GFP‐RFP‐LC3 dual fluorescence assay showing reduced autophagosome‐lysosome fusion (red puncta indicated by white arrows, Figure 6c), collectively demonstrating impaired autophagic flux. Crucially, 3‐MA suppressed the upregulation of DSPP, DMP‐1, Osx, and Runx2 mRNA and protein expression levels triggered by 0.1 μg/mL LPS (Figure 6d,e), concomitant with diminished mineralisation capacity (Figure 6f).

These data conclusively establish that functional autophagic flux is indispensable for odontogenic differentiation and mineralisation in hDPCs under inflammatory stress. Enhancing autophagy with rapamycin or inhibiting it with 3‐MA directly affects the odontogenic capacity of cells. This demonstrates that autophagy is a viable therapeutic target to counteract inflammation‐related damage in pulp repair.

### 3.5 Cx43 Inhibition Enhanced Autophagic Flux to Potentiate Odontogenic Differentiation of hDPCs Under High‐Inflammatory Conditions

**FIGURE 7:**
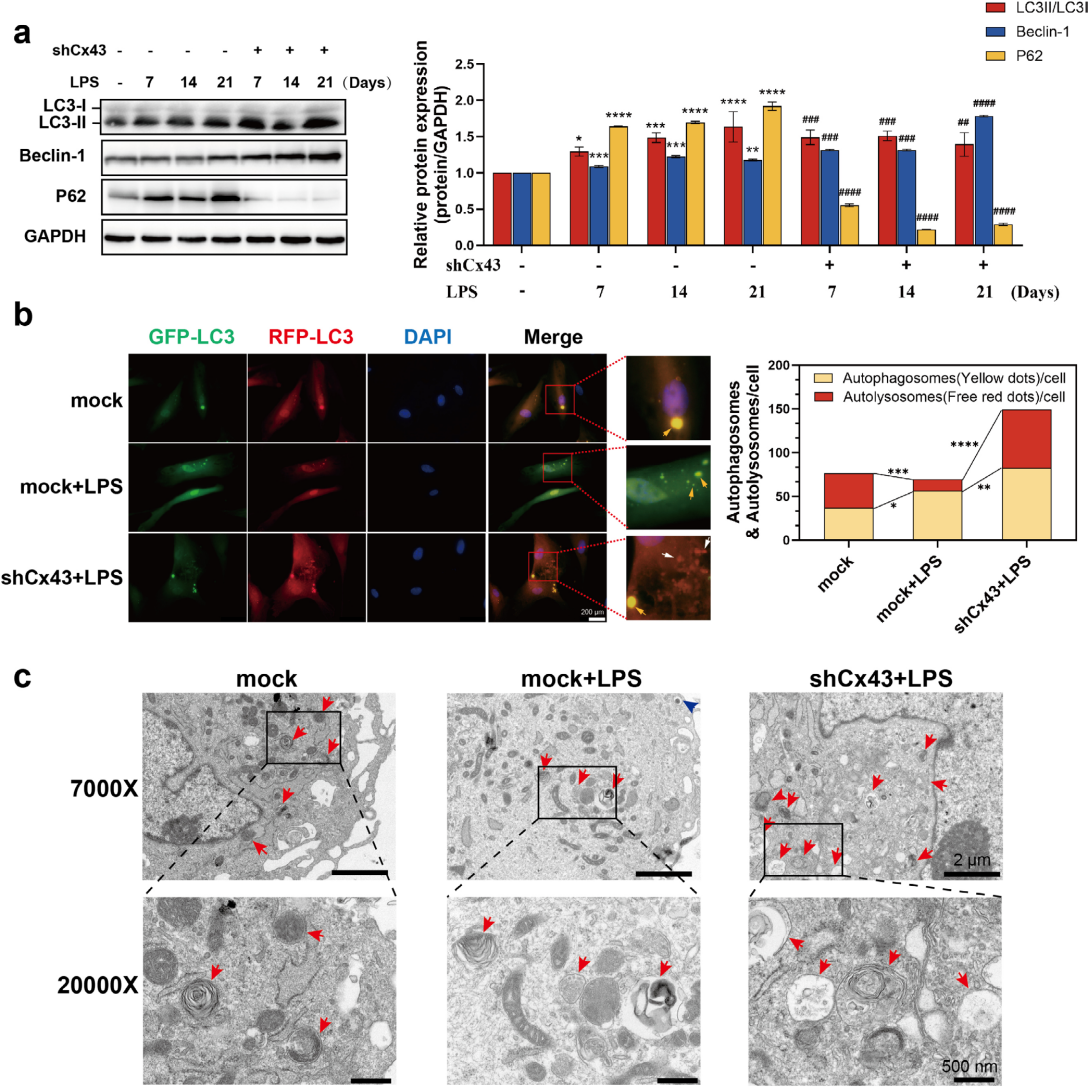
Cx43 inhibition enhances autophagic flux in hDPCs. hDPCs transfected with shRNA‐Cx43 (shCx43) or shRNA‐mock (mock) were stimulated with OI + 5 μg/mL LPS. (a) WB analysis of LC3II/I ratio, Beclin‐1, and p62. (b) mRFP‐GFP‐LC3 fluorescence imaging. (c) TEM analysis of autophagic ultrastructure showing autolysosomes (red arrows). *p < 0.05, **p < 0.01, ***p < 0.001, ****p < 0.0001 versus mock; ^##^ *p* < 0.01, ^###^ *p* < 0.001, ^####^ *p* < 0.0001 versus mock + LPS; *p < 0.05, **p < 0.01, ***p < 0.001, ****p < 0.0001 in (b).

Emerging evidence indicates that Cx43 exerts dual regulatory control over autophagy through: (1) direct protein–protein interactions with core autophagy machinery (e.g., LC3) to govern autophagosome formation (Iyyathurai et al. 2016; Bejarano et al. 2014), and (2) GJ channel activity‐dependent modulation of autophagic flux (Zou et al. 2020). Our previous studies have independently demonstrated two context‐dependent functions of Cx43 in dental pulp biology: (1) Hemichannel blockade attenuates LPS‐induced inflammatory responses by limiting DAMPs efflux (Hu et al. 2024), while (2) GJ channel potentiation enhances Ca^2+^‐mediated the odontogenic differentiation through Erk1/2 signalling (Li et al. 2015). Thus, we hypothesised that Cx43‐mediated autophagy regulation might orchestrate odontogenic differentiation. In the current investigation, inhibition of Cx43 in LPS‐challenged (5 μg/mL) hDPCs significantly elevated LC3II/I ratios and Beclin‐1 expression levels while suppressing p62 accumulation (Figure 7a). In line with these biochemical alterations, Cx43 suppression augmented autophagosome‐lysosome fusion as evidenced by GFP‐RFP‐LC3 reporter‐based immunofluorescence (red puncta indicated by white arrows, Figure 7b), while TEM structurally validated this enhancement via quantification of autophagic vacuoles with lysosomal characteristics (red arrows, Figure 7c). The coordinated elevation in fusion frequency across both imaging modalities established Cx43 inhibition as a regulator of autophagic flux progression in hDPCs.

**FIGURE 8:**
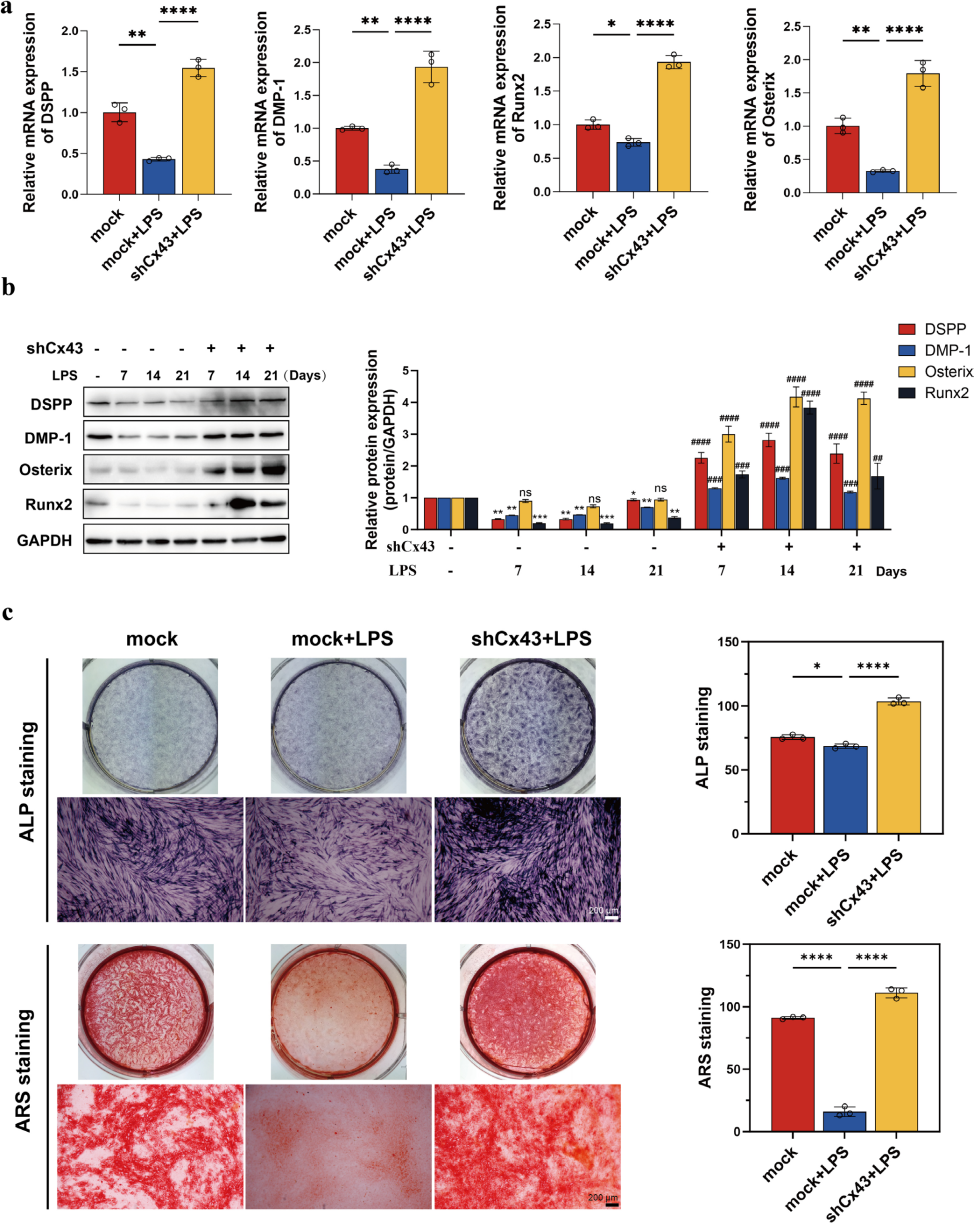
Cx43 inhibition promotes odontogenic differentiation in LPS‐stimulated hDPCs. (a) RT‐qPCR analysis of odontogenic markers. (b) WB analysis of corresponding proteins. (c) ALP activity and mineralisation. *p < 0.05, **p < 0.01, ****p < 0.0001 in (a, c); *p < 0.05, **p < 0.01, ***p < 0.001 versus mock in (b); ^##^ *p* < 0.01, ^###^ *p* < 0.001, ^####^ *p* < 0.0001 versus mock + LPS in (b).

Furthermore, Cx43 inhibition upregulated both mRNA and protein expression levels of odontogenic markers in 5 μg/mL LPS‐challenged hDPCs (Figure 8a,b). Functional validation revealed concomitant enhancement of ALP activity and mineralised nodule formation (Figure 8c), suggesting that Cx43 modulation critically regulates the inflammatory microenvironment‐compromised odontogenic differentiation. These coordinated findings in vitro position autophagic flux as a pivotal mechanistic link between Cx43 activity and differentiation potential under high‐inflammatory conditions.

**FIGURE 9:**
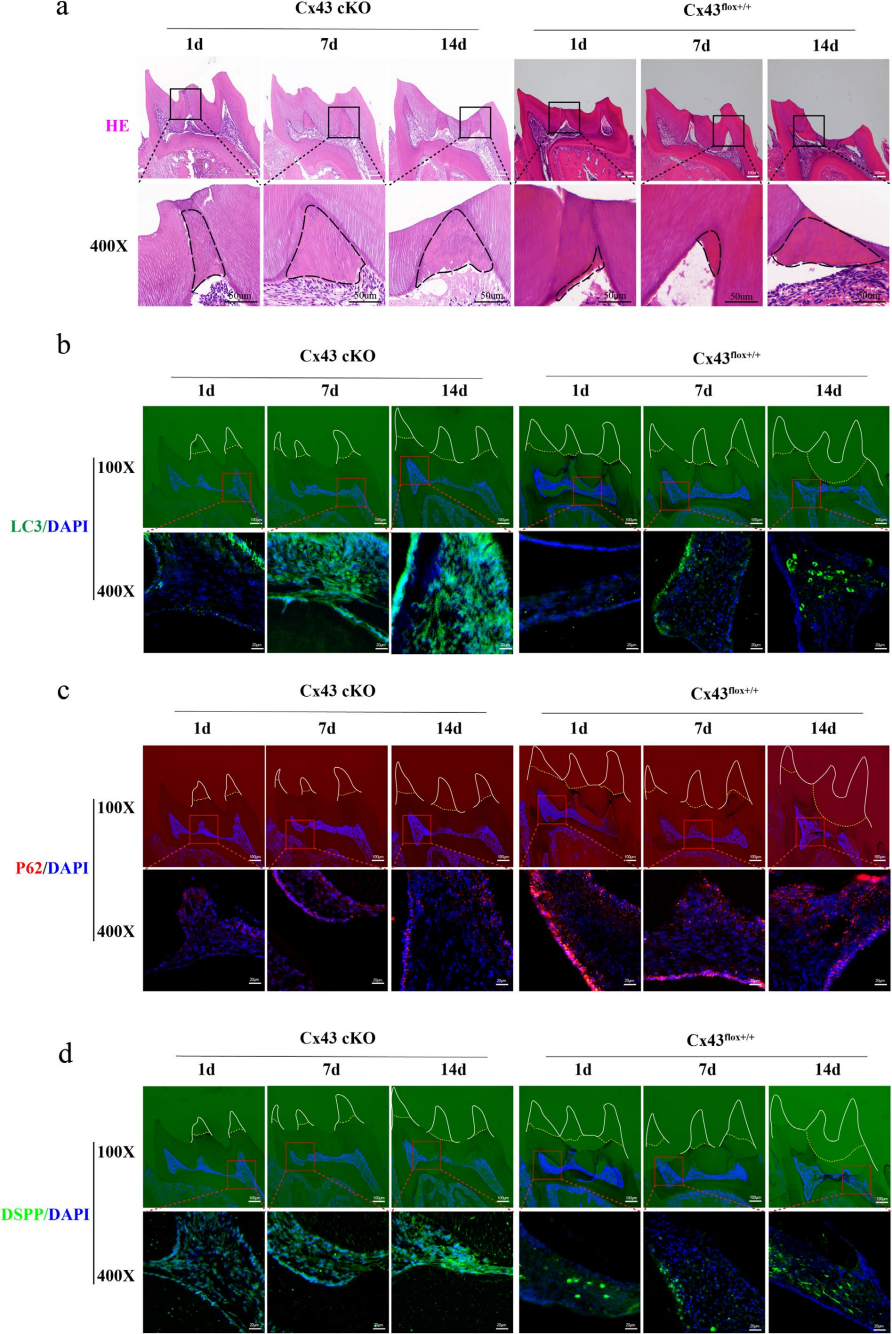
Cx43 conditional knockout enhances autophagic flux and tertiary dentine formation. (a) Haematoxylin and eosin staining of tertiary dentine (black boxes) in Cx43^flox+/+^ and cKO mice at days 1, 7, and 14 post‐injury. (b–d) Fluorescence intensity of LC3, p62, and DSPP. white boxed area indicates ground‐down dental tissue.

To establish clinical relevance, we utilised dental pulp‐specific Cx43‐cKO mice (driven by the DSPP promoter‐Cre) in a maxillary first molar dentine injury model. Notably, cKO mice exhibited accelerated tertiary dentine formation compared to the Cx43^flox+/+^ littermates, with mineralised matrix deposition (black boxes) increasing at days 7 and 14 post‐injury, respectively (Figure 9a). Spatiotemporal analysis revealed: (1) IF‐based LC3 signal intensification subjacent to injury sites at day 7 and 14 compared to the control (Figure 9b); (2) Concomitant p62 downregulation (Figure 9c); (3) Upregulated DSPP expression paralleling repair progression (Figure 9d). This multimodal in vivo evidence not only recapitulates our findings in vitro but also establishes the Cx43‐autophagy axis as a conserved regulator of pulp‐dentine complex regeneration under pro‐inflammatory conditions. Collectively, our integrated experimental approach spanning molecular profiling, functional assays, and transgenic models reveals a previously unappreciated Cx43 gatekeeper function: through autophagic flux modulation, it dynamically calibrates odontogenic differentiation capacity in inflammatory milieus, ultimately dictating dental pulp repair outcomes.

## 4 Discussion

**FIGURE 10:**
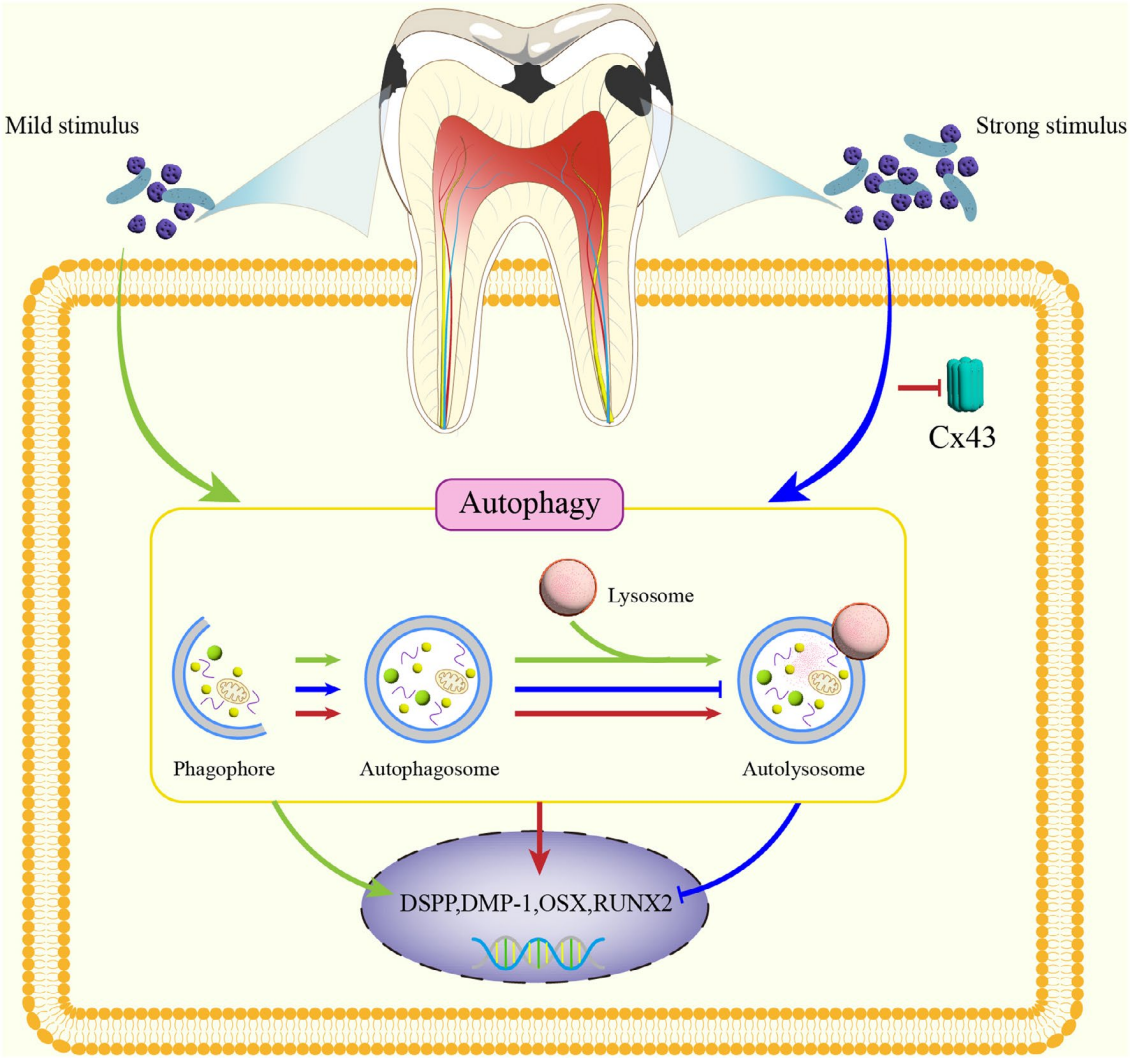
Proposed mechanism of autophagy and Cx43 in pulp repair under inflammation. Three pathways mediate inflammation‐driven odontogenic differentiation (colour‐coded): Green pathway: Moderate stimulation (e.g., enamel caries) induces low‐grade inflammation, which activates autophagy and sustains autophagic flux, thereby upregulating odontogenic differentiation to facilitate tertiary dentine formation. Blue pathway: High‐grade inflammation (induced by strong stimulus, such as deep dentine caries) induces autophagosome formation but blocks autophagosome‐lysosome fusion, impairing autophagic flux and dentine repair. Red pathway: Cx43 inhibition under high‐grade inflammation rescues autophagic flux, restoring odontogenic differentiation and dentine regeneration.

In this study, we found that autophagy was activated in inflamed dental pulp, and the level of autophagy changed with the severity of infection. This study clarifies the role of autophagy and autophagy flux in odontogenic differentiation and also elucidates the critical mechanism by which Cx43 regulates odontogenic differentiation of hDPCs in a high‐concentration LPS‐induced inflammatory environment through modulation of autophagic flux (The hypothetical mechanism diagram is presented in Figure 10).

The cellular degradative pathway of autophagy has a fundamental role in immunity and inflammation, which the impairment of autophagy can mediate susceptibility to infectious and inflammatory diseases (Deretic 2021; Matsuzawa‐Ishimoto et al. 2018). In the context of dental pulp tissue, LC3 is observed in human teeth with deep caries and pulpitis, indicating that autophagy is activated in inflamed human dental pulp (Wang et al. 2016). Our results in human third molars demonstrate that autophagy is not only activated within the dental pulp but also enhanced with increasing severity of infection, indicating that autophagy is involved in the infection and inflammation of dental pulp. Furthermore, the expression of p62 was gradually upregulated in dental pulp, suggesting that autophagic flux may be inhibited with the progression of the disease.

Pei et al. found that autophagy in vitro was induced by LPS in the pre‐odontoblast cell line mDPC6T, which played a dual role in cell survival (Pei et al. 2015). A recent study showed that the promotion of autophagy by rapamycin could decrease *P. gingivalis* ‐induced the inflammatory response in human dental pulp fibroblasts (Feng et al. 2024). Meanwhile, autophagy was shown to regulate the cell activity of odontoblasts for dentine formation (Park et al. 2021, 2020) and promote odontogenic differentiation of hDPCs under physiological conditions (Cho et al. 2021; Park et al. 2020). Moreover, autophagy could promote odontogenic differentiation of mouse dental papilla cells in an inflammatory environment by suppressing NF‐κB (Pei et al. 2016), which serves as a pivotal mediator of inflammatory response (Barnabei et al. 2021). Growing evidence clearly indicates that dentine barrier formation only occurs when pulp infection and inflammation are diminished, thus enabling the reestablishment of tissue homeostasis and health (Farges et al. 2015; Cooper et al. 2014, 2017, 2010; Zaky et al. 2021). Thus, it can be inferred that autophagy may promote odontogenic differentiation and tissue repair by inhibiting pulp inflammation.

In this study, we found that low‐concentration LPS (0.1 μg/mL) enhanced the odontogenic differentiation in hDPCs by promoting autophagic flux (increased autophagosome‐lysosome fusion). This aligns with Pei et al. (2016), who proposed that autophagy facilitates differentiation by suppressing NF‐κB. Moreover, Zhan et al. also found that inhibition of autophagic flux decreased autophagy activity, which led to down‐regulation of odontoblast differentiation (Zhan et al. 2020). The integrity of autophagic flux likely maintains intracellular homeostasis by clearing inflammation‐induced damaged proteins (e.g., ubiquitinated NF‐κB), thereby providing energy and substrates for differentiation. Conversely, high‐concentration LPS (5 μg/mL), despite inducing autophagosome formation (upregulated LC3‐II/I and Beclin‐1), disrupted autophagic flux (p62 accumulation, reduced lysosomal fusion), leading to impaired differentiation. This supports Pellegrini et al. (2023), who hypothesised that autophagic flux blockage triggers cellular stress to inhibit cell activity and promote apoptosis‐mediated cell death associated with reactive oxygen species (ROS) production. In the context of inflamed hDPCs, we propose two potential mechanisms for this disruption: (1) LPS activates ROS via Toll‐like receptor (TLR)‐4 to inhibit V‐ATPase function, thereby impairing lysosomal acidification (Sanjuan et al. 2007; Fu, Chen, et al. 2024). (2) Pro‐inflammatory cytokines induced by LPS downregulate Ras‐related Protein Rab‐7 (Rab7) or Lysosome‐Associated Membrane Protein 1 (LAMP1) expression, leading to autophagosome‐lysosome fusion defects (Thurston et al. 2009; Zeng et al. 2022). Anyway, these findings underscore that the completeness of autophagic flux rather than mere autophagosome formation is pivotal for odontogenic differentiation of hDPCs under inflammatory conditions.

Interestingly, a recent study suggested that autophagy is involved in the suppression of stem cells from apical papilla (SCAP) osteo/odontogenic differentiation in 5 μg/mL LPS‐induced inflammatory environment (Lei et al. 2020), which seems to be contrary to our results. However, there are some differences between two research studies. (1) That literature focuses on autophagosome formation but does not explicitly evaluate the completion of autophagic flux. High concentrations of LPS (5 μg/mL) may induce excessive autophagosome formation without effective degradation (impaired autophagic flux), leading to an imbalance in cellular homeostasis and inhibition of differentiation. Our findings clarify the impairment of autophagic flux under high LPS concentrations through accumulation of p62, GFP‐RFP‐LC3 tandem fluorescent assay, and TEM observation. However, by enhancing autophagic flux with rapamycin (Zhou et al. 2012), we restore the integrity of autophagic flux, thereby reversing differentiation inhibition. The dynamic balance of autophagic flux (rather than autophagosome formation alone) is crucial for differentiation. (2) That literature employs SCAP, whose biological behaviours (such as differentiation potential and inflammatory response) may differ from those of dental pulp‐derived cells (Tziafas and Kodonas 2010; Smeda et al. 2022). (3) Our findings distinguish between the effects of low‐concentration (1 μg/mL) and high‐concentration (5 μg/mL) LPS. Low‐concentration LPS enhances differentiation by promoting autophagic flux, whereas high‐concentration LPS inhibits differentiation due to impaired autophagic flux. The direction of differentiation is determined by the integrity of autophagic flux through inflammatory intensity. That literature mainly focuses on the inhibitory effect of high‐concentration LPS without exploring the potential promotive effect at low concentrations or differentiating the dynamic changes in autophagic flux.

Cx43, a gap junction protein, is known to regulate a wide range of cellular processes via its channel and non‐channel functions, including inflammation, cell differentiation, and survival (Su and Lau 2012). Studies have shown that Cx43 may be involved in the maintenance of odontoblast arrangement patterns and influence the pulp repair outcomes by the regulation of odontogenic differentiation (Yin et al. 2021). Our previous studies have revealed that Cx43 exerts dual therapeutic effects. It not only mitigates 5 μg/mL LPS‐induced inflammation in hDPCs through hemichannels by inhibiting extracellular flux of DAMPs (Hu et al. 2024) but also promotes odontogenic differentiation of dental pulp stem cells via GJ channels (Li et al. 2015). These findings collectively indicate that Cx43 plays a critical role in coordinating inflammatory responses and facilitating reparative processes in dental pulp tissue. Groundbreaking studies have implicated that Cx43 has the capacity to bind to autophagy‐related proteins to regulate autophagy processes (Bejarano et al. 2014; Martins‐Marques et al. 2019). Moreover, recent data have indicated that gap junctions containing Cx43 are also implicated in the regulation of autophagy and even influence autophagic flux, thereby affecting cellular function (Zou et al. 2020; Duan et al. 2023). In this study, silencing Cx43 promoted autophagic flux and upregulated the odontogenic markers in hDPCs under inflammatory conditions. In vivo validation using Cx43 cKO mice confirmed decreased p62 and enhanced DSPP co‐localization post‐injury. Specifically, we found that knocking down Cx43 expression reversed the inhibitory effect of high‐level inflammation on autophagic flux and odontogenic differentiation, highlighting Cx43's role as a negative regulator of autophagic flux and dental pulp repair under inflammatory stress. The underlying molecular mechanisms may be that (1) Channel‐dependent pathway: Cx43 hemichannels released DAMPs (e.g., ATP, HMGB1) (Hu et al. 2024), activating NOD‐like receptor family pyrin domain containing 3 (NLRP3) inflammasomes and indirectly suppressing lysosomal function (Biasizzo and Kopitar‐Jerala 2020; Kelley et al. 2019). (2) Non‐channel‐dependent pathway: the Cx43 C‐terminus directly interacted with Beclin‐1 or VPS34 complexes, interfering with autophagosome maturation (Bejarano et al. 2014). The observed Beclin‐1 upregulation upon Cx43 knockdown in Figure 7a suggested competitive binding to autophagy‐related proteins.

The translational strength of this study is to provide valuable insights into potential therapeutic strategies for promoting pulp regeneration in inflammatory conditions. By modulating Cx43 expression or enhancing autophagic flux, it may be possible to reverse the inhibitory effects of inflammation on pulp cell function and promote odontogenic differentiation and mineralisation after removing infection. Thus, developing Cx43‐targeted therapeutic agents, such as TAT‐Gap19 (hemichannel inhibitor) or Cx43 C‐terminal mimetic peptides, could restore autophagic flux to promote dentine regeneration. However, this study has limitations in the mechanistic exploration of Cx43‐mediated regulation, as specific interacting partners and downstream signalling pathways were not fully characterised. Furthermore, current rodent molar injury models fail to recapitulate the chronic inflammatory microenvironment in human progressive pulpitis, necessitating more clinically relevant preclinical systems. Future research should focus on two complementary directions: (1) Utilising co‐culture systems simulating macrophage‐dental pulp cell interactions to dissect the immunomodulatory dynamics of the inflammation‐autophagy‐differentiation axis (Gopinath et al. 2023). (2) Deciphering Cx43's interaction network with autophagy core proteins to advance precision therapeutic strategies.

In conclusion, our study unveils a novel mechanism by which Cx43 governs odontogenic differentiation of hDPCs through autophagic flux modulation in inflammatory environments, establishing the ‘inflammation‐autophagy‐differentiation axis’ as a key target for pulp regeneration.

## Author Contributions

Shiting Li designed the study. Peiling Hu, Yingqing Chen, and Ping Long conducted the experiments. Xiaorong Lan and Yuanpei He analysed the data. Guangwen Li supervised the project. Anni Zhang wrote the paper. All authors commented on the paper and approved this manuscript.

## Ethics Statement

The protocol was approved by the Ethics Committee of the Affiliated Stomatology Hospital of Southwest Medical University (Reference: 20180511001).

## Conflicts of Interest

The authors declare no conflicts of interest.

## Supporting information


**Data S1:** iej70044‐sup‐0001‐DataS1.docx.

## Data Availability

Data available on request from the authors.
