# Supplementary material for: From Cells to Animals: Connexin43 Suppression Enhances Autophagic Flux to Restore Odontogenesis in Inflamed Dental Pulp
Source: Int Endod J. 2025 Oct 5;59(1):134–52. doi: 10.1111/iej.70044 (PMC12701752; doi:10.1111/iej.70044)
Supplement: Supplementary file 1 — Data S1: iej70044‐sup‐0001‐DataS1.docx. [file IEJ-59-134-s001.docx]

**Generation of Hipp11 Knock-in Mice**

We established a conditional knockout mouse model in the C57BL/6N background targeting the *Gja1* gene (NCBI ID: 14609). **Conditional Knockout Strategy**: LoxP sites were inserted flanking the exon of *Gja1* to enable tissue-specific deletion via Cre recombinase (Fig. S1a). **F1 Generation PCR Screening**: Genotyping of F1 offspring (IDs 1, 2, 6, and 9) was performed using two primer pairs (Primer 1: F-5'-TTCAGAGTAAAACTGGTCTAGCCT-3', R-5'-GTCTGTATGCCTCTAAGCAAAACG-3', amplicon size: 216 bp; Primer 2: F-5'-GGCCTGATGACCTGGAGATTTA-3', R-5'-ATACCGTTACATACCTCCCCTTT-3', amplicon size: 280 bp). All four mice showed positive bands, confirming successful heterozygous knock-in (Fig. S1b).


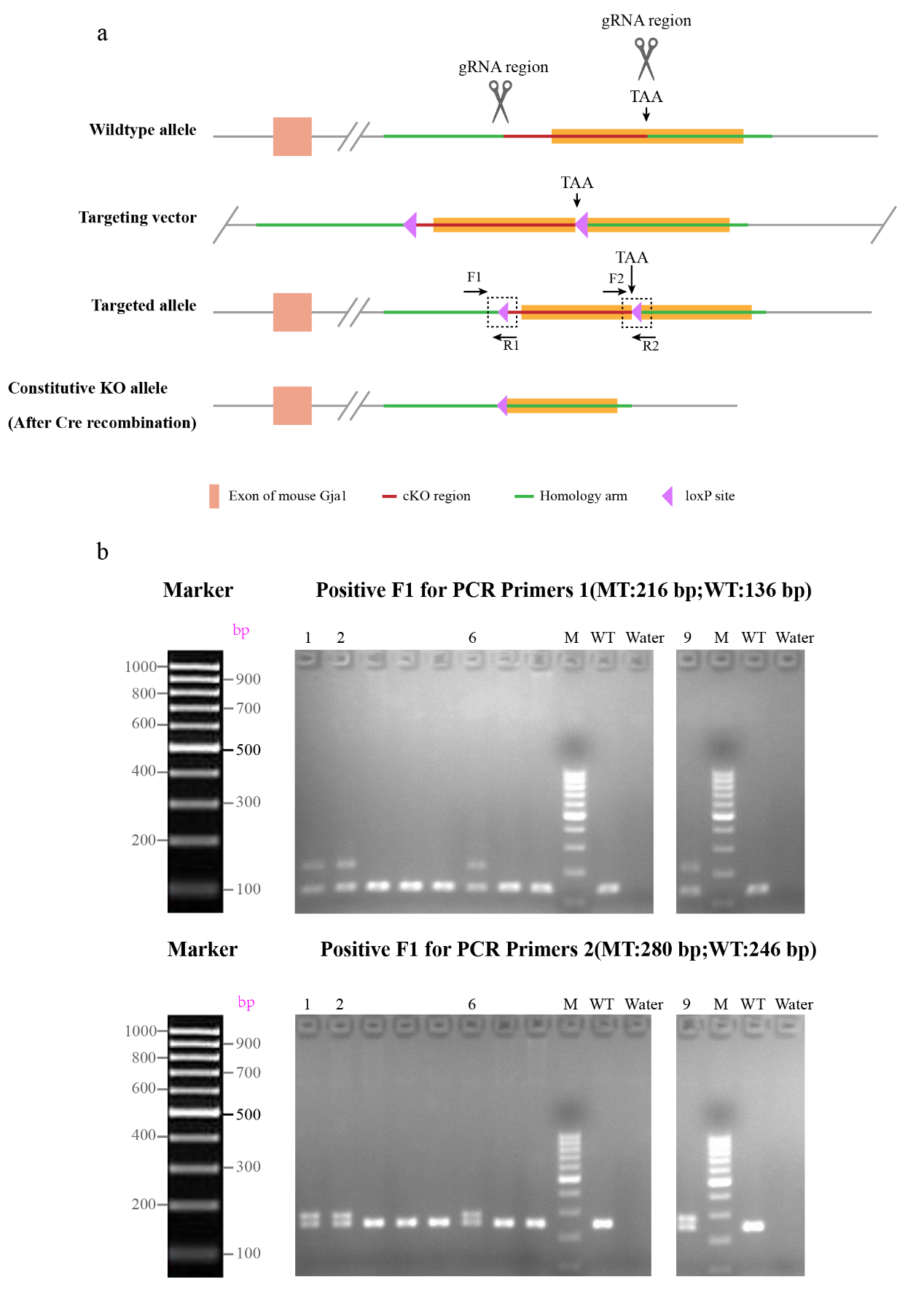


**Fig. S1 the generation of Cx43^flox+/-^ mice**. **a** Enotyping strategy. **b** F1 animals 1, 2, 6, and 9 were identified positive by PCR screening.

**Construction of DSPP promoter-CreERT^+^ Mice**

The "Mouse Dspp promoter-Kozak sequence-CreERT2-rBG polyA" cassette was precisely knocked into the Hipp11 (H11) locus using CRISPR/Cas9 technology (genotyping strategy in Fig. S2a). **Microinjection and Identification**: Fertilized mouse eggs were co-injected with H11-targeting guide RNA (sequence: GAACACTAGTGCACTTATCCTGG), Cas9 mRNA, and donor plasmids to generate F0 founders. F0 founders were subsequently crossed with wild-type C57BL/6 mice to screen for germline transmission. F1 founders were validated by PCR (Primer 1: F-5'-GTGGGCATTGGTTATTGGTCGTAG-3', R-5'-TAATCGCGAACATCTTCAGGTTCT-3', amplicon size: 3.7 kb; Primer 2: F-5'-GCTCTACTTCATCGCATTCCTT-3', R-5'-TGTGAGTCACCACGCTTGCCTTG-3', amplicon size: 2.9 kb; Fig. S2b) followed by sequence analysis (Fig. S2c), which were bred to wildtype mice to test germline transmission and F1 animal generation. **Southern Blot Validation**: Tail DNA from F1 mice was digested with BstEII or SspI and hybridized with 5' probes (5'-GGCACAATGTTAATCCAGCCTGACTC-3' and 5'-GTGACCAGTTTGTCCTCCTCCAGTAGA-3') and 3' probes (5'-GATGTGAACAAAGCACCCTATGGCTC-3' and 5'-GTGTCGATCATCCATTAGCCTAGCC-3'), respectively. Expected fragments (WT: 11.35 kb/4.12 kb; mutant: 14.73 kb/5.46 kb) confirmed site-specific integration (Fig. S2d). **Homozygote Breeding Strategy**: Heterozygous targeted mice were intercrossed to generate homozygous targeted mice. Short PCR primers (wildtype: F-5'-CTCTACTGGAGGAGGACAAACTG-3', R-5'-GTCTTCCACCTTTCTTCAGTTAGC-3', amplicon size: 519 bp; mutant: F-5'-GCTCTACTTCATCGCATTCCTT-3', R-5'-CTTTATTAGCCAGAAGTCAGATGC-3', amplicon size: 237 bp) were designed to distinguish homozygotes from wildtype (Fig. S2e).


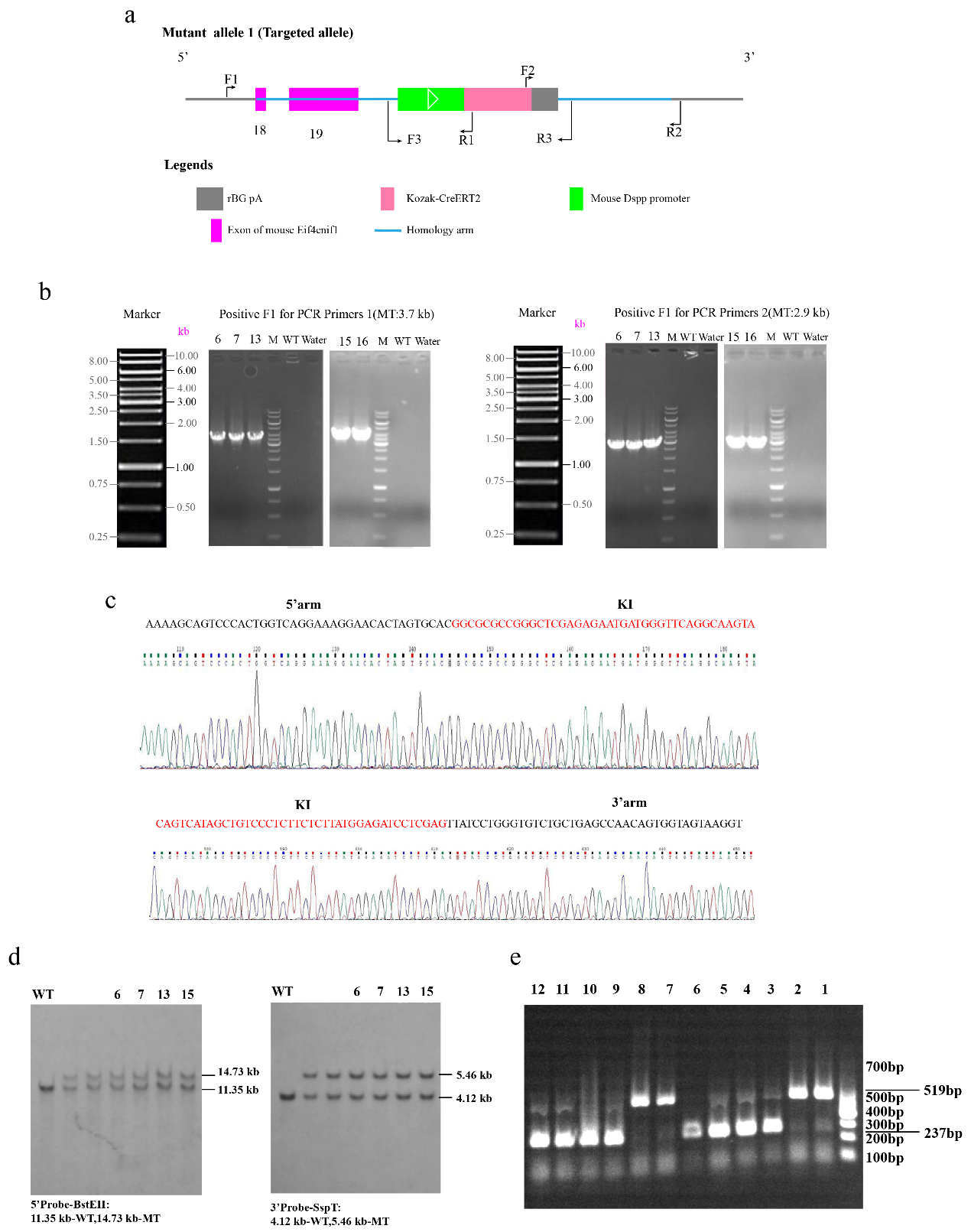


**Fig. S2 the construction of DSPP promoter-CreERT^+^ mice**. **a** Genotyping strategy. **b** F1 animals 6, 7, 13, and 15 were identified positive by PCR screening. **c** Sequencing confirming for mouse ID: 6 (one example of positive mice). **d** The correct gene targeting in 4 F1 animals (6, 7, 13, and 15) were confirmed by southern blot analysis of the tail DNA samples. **e** Inter-cross heterozygous targeted mice to generate homozygous targeted mice. Animals 3, 4, 5, 9, 10, 11, and 12 were identified positive by PCR screening.

**Generation of DSPP promotor-Cre; Cx43^flox+/+^ Mice**

**Breeding Strategy** (Fig. S3a): Cx43^flox+/-^ male heterozygotes were crossed with wildtype B6 mice to generate Cx43^flox+/+^ offspring. Genotyping of 3-4-week-old mice was performed using tail DNA and primers (F-5'-TTCAGAGTAAAACTGGTCTAGCCT-3', R-5'-GTCTGTATGCCTCTAAGCAAAACG-3'; amplicon size: 216 bp; Fig. S3b). Cx4^flox+/-^ males were then bred with CreERT^+^ mice to obtain CreERT^+^; Cx43^flox+/-^ progeny. Subsequent intercrosses yielded CreERT^+^; Cx43^flox+/+^ mice, validated by PCR (F-5'-GGCCTGATGACCTGGAGATTTA-3', R-5'-ATACCGTTACATACCTCCCCTTT-3'; amplicon size: 330 bp; Fig. S3c). **Tamoxifen Induction**: Four-week-old mice received intraperitoneal tamoxifen (75 mg/kg) for 5 consecutive days. Seven days post-induction, dental pulp tissues were harvested for RIPA lysis and Western blotting (WB) to confirm Cx43 depletion (Fig. S3d).


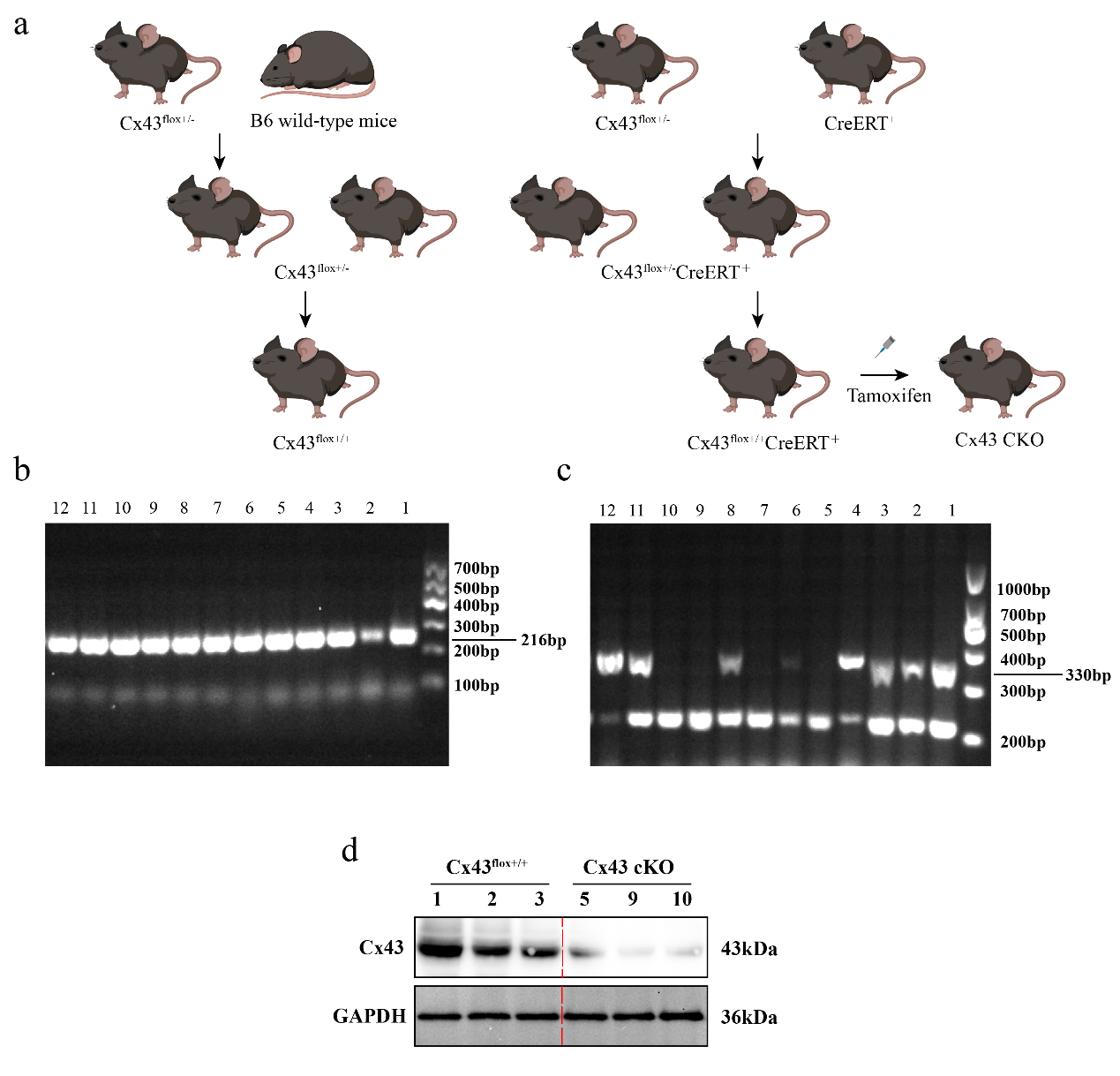


**Fig. S3 the generation of DSPP Pro-Cre; Cx43^flox+/+^ mice**. **a** Protocol for establishing a conditional knockout mouse line via crossing with CreERT mice. **b** Cx43^flox+/+^ mice were identified positive by PCR screening. **c** CreERT^+^; Cx43^flox+/+^ mice were identified positive by PCR screening. **d** Determining Cx43 expression in dental pulp of cKO mice via WB.
